# Association Between Oral Microbiota and Oral Leukoplakia: A Systematic Review

**DOI:** 10.7759/cureus.52095

**Published:** 2024-01-11

**Authors:** Nishath Sayed Abdul, Yara Rashdan, Najd Alenezi, Muneera Alenezi, Lamia Mohsin, Aldanah Hassan

**Affiliations:** 1 Department of Oral and Maxillofacial Surgery and Diagnostic Sciences, Faculty of Oral Pathology, College of Medicine and Dentistry, Riyadh Elm University, Riyadh, SAU; 2 College of Medicine and Dentistry, Riyadh Elm University, Riyadh, SAU

**Keywords:** dysbiosis, oral squamous cell carcinoma, pre-malignant disorders, leukoplakia, oral microbiome

## Abstract

Literature evidence suggests a significant gap in research exploring the association between the oral microbiota and leukoplakia. So, this review aimed to thoroughly assess the body of research and look at the connection between leukoplakia and the oral microbiome. Databases such as Pubmed/MEDLINE (Medical Literature Analysis and Retrieval System Online), Embase (Excerpta Medica Database), Web of Science, Scopus, CINAHL (Cumulated Index to Nursing and Allied Health Literature), and Cochrane Library were searched using MeSH keywords using a standard data extraction protocol that was designed to ensure comprehensive extraction of relevant information from the selected studies, adhering to the PRISMA (Preferred Reporting Items for Systematic Reviews and Meta-Analyses) guidelines. Seven studies were selected that were relevant to this review’s objectives. The findings indicate that patients with leukoplakia had a diverse oral microbiota compared to healthy controls. The connection between the oral microbiomes of leukoplakia patients and oral cancer cases was also found, indicating possible microbial profile similarities. By shedding light on particular microbial species and variations in the oral microbial flora of leukoplakia, the studies that were included in this review reveal possible biomarkers and provide a deeper knowledge of the disease mechanism. The considerable overlap between oral microbiomes in leukoplakia cases highlights the need for more research into common microbial indicators and the implications for diagnosis and prognosis.

## Introduction and background

The oral microbiome represents the complex and diverse stratification of microorganisms inhabiting the oral cavity [1]. It is composed of bacteria, fungi, and other microorganisms that co-exist in symbiosis with the human individual [2]. It is highly dynamic and influenced by several variables such as dietary factors, oral hygiene practices, genetics, and environmental factors [2].

The oral microbiome has an important role in oral health maintenance and homeostasis. It helps in the digestion of certain dietary components, contributes to the formation and maintenance of dental structures, and assists in developing and regulating the immune system [1]. Additionally, the oral microbiome helps to prevent the colonization of pathogenic microbes by competing for resources and producing antimicrobial products [3].

Advancements in advanced DNA sequencing technologies have enabled the characterization and understanding of the oral microflora in greater detail. This allows researchers to identify specific microbial species and their interactions within the oral ecosystem [4]. Understanding the composition, diversity, and functioning can provide valuable insights into disease mechanisms, risk assessment, and the development of novel strategies for oral health maintenance and treatment [5].

Leukoplakia is characterized by the formation of whitish or grayish patches on the mucosal membranes of the mouth, including the tongue, cheeks, gingiva, and floor of the mouth [6]. These patches are usually painless and have a thick, hardened, or rough texture. The exact etiological factor or role of leukoplakia is not fully understood, but it is commonly linked with chronic irritation or inflammation of the dental mucosa [7]. Chronic tobacco use, both smoking and smokeless forms, is considered a major risk factor for leukoplakia. Other factors that may contribute to its development include alcohol consumption, poor oral hygiene, chronic irritation from rough or ill-fitting dental restorations, and some viral infections, such as human papillomavirus (HPV). While most cases of leukoplakia are benign, some lesions may have the potential for malignant transformation into squamous cell carcinoma (oral cancer), especially in long-standing or dysplastic leukoplakia [6]. Therefore, it is essential for individuals with leukoplakia to undergo regular monitoring and evaluation by a healthcare professional.

There are several literature gaps and areas for further investigation regarding the correlation between the oral microbiome and leukoplakia. For example, longitudinal studies with well-defined cohorts and extended follow-up periods are needed to understand the temporal dynamics and causality between changes in the oral microbiome and the development and progression of leukoplakia [8-9]. The review aimed to summarize and synthesize the available evidence to determine whether there is a consistent association between alterations in the oral microbiome and the presence, development, or progression of leukoplakia.

## Review

Methods

Review Protocol

This review adhered to the lines of Preferred Reporting Items for Systematic Reviews and Meta-Analyses (PRISMA) guidelines [10-11].

PECO Strategy

The PECO (Population, Exposure, Comparator, Outcome) strategy for this investigation is given below.

Population: It encompasses human individuals diagnosed with oral leukoplakia. This includes both genders, all age groups, and various ethnic backgrounds.

Exposure: The subject of interest was the composition of the oral microbiota. This was assessed employing microbiological techniques and metagenomic sequencing, capturing the microbial diversity and abundance within the oral cavity.

Comparator: The comparator in this review is healthy controls or the human population without leukoplakia.

Outcome: The outcome of interest was the association between oral microbiota and oral leukoplakia. This encompassed examining whether specific microbial species, diversity indices, or compositional changes in the oral microbiota were associated with the occurrence, severity, or progression of oral leukoplakia.

Search Strategy

An exhaustive search was conducted to retrieve relevant studies from databases such as Pubmed/MEDLINE (Medical Literature Analysis and Retrieval System Online, Embase (Excerpta Medica Database), Web of Science, Scopus, CINAHL (Cumulated Index to Nursing and Allied Health Literature), and the Cochrane Library, as shown in Table 1. The Boolean operator "AND" was used to ensure that both terms related to oral microbiota and oral leukoplakia were present in the retrieved studies. The operator "OR" was employed to combine synonyms or related terms within the same concept, expanding the search scope.

**Table 1:** Search strategy employed MEDLINE: Medical Literature Analysis and Retrieval System Online; Embase: Excerpta Medica Database; CINAHIL: Cumulated Index to Nursing and Allied Health Literature

Database	Search Strategy
PubMed/MEDLINE	("Oral Microbiota"[Mesh] OR "Mouth Microbiota"[Mesh] OR "Microbiota"[Mesh]) AND ("Leukoplakia, Oral"[Mesh] OR "Oral Leukoplakia"[Mesh] OR "Leukoplakia"[Mesh])
Embase	('oral microbiota'/exp OR 'mouth microbiota'/exp OR 'microbiota'/exp) AND ('oral leukoplakia'/exp OR 'leukoplakia, oral'/exp OR 'leukoplakia'/exp)
Web of Science	TS=("oral microbiota" OR "mouth microbiota" OR "microbiota") AND TS=("leukoplakia, oral" OR "oral leukoplakia" OR "leukoplakia")
Scopus	TITLE-ABS-KEY("oral microbiota" OR "mouth microbiota" OR "microbiota") AND TITLE-ABS-KEY("leukoplakia, oral" OR "oral leukoplakia" OR "leukoplakia")
CINAHL	(MH "Oral Microbiota" OR MH "Mouth Microbiota" OR MH "Microbiota") AND (MH "Leukoplakia, Oral" OR MH "Oral Leukoplakia" OR MH "Leukoplakia")
Cochrane Library	("Oral Microbiota"[Mesh] OR "Mouth Microbiota"[Mesh] OR "Microbiota"[Mesh]) AND ("Leukoplakia, Oral"[Mesh] OR "Oral Leukoplakia"[Mesh] OR "Leukoplakia"[Mesh])

To enhance the sensitivity of the search, additional terms were included to capture various aspects of the topic. These terms included synonyms and related concepts for oral microbiota (e.g., "oral flora," "oral microbial community") and oral leukoplakia (e.g., "oral white patches," "oral mucosal hyperkeratosis"). Truncation and wildcard symbols were employed where applicable to capture different word endings and variations.

Selection Criterion

The eligibility criteria were defined priorly and applied during the study selection process to maintain methodological rigor. Original research published in peer-reviewed journals was included. Studies involving humans with a definitive diagnosis of oral leukoplakia were included. There were no restrictions on participant characteristics such as age, gender, or ethnicity. The review was limited to English-language publications due to resource constraints and language proficiency. Conference abstracts, theses, dissertations, and grey literature were excluded to maintain the quality and reliability of the evidence. Studies conducted on animals or using in vitro models were excluded, as the focus of this review was on human studies. Those not reporting relevant outcomes of our interest were excluded.

Data Extraction

A detailed data extraction form was developed, which included specific fields to capture pertinent information. The form encompassed key aspects such as study characteristics (e.g., authors, publication year, study design), participant characteristics (e.g., sample size, demographics), exposure assessment (e.g., methods used to assess oral microbiota), and outcome measures. To ensure consistency and minimize bias, a calibration exercise was conducted among the reviewers. This exercise involved jointly extracting data from a subset of included studies and discussing any discrepancies or uncertainties to establish consensus and enhance inter-reviewer agreement. Once the calibration exercise was completed, the actual data extraction process commenced. Each reviewer independently extracted data from the assigned studies. Throughout the process, regular meetings were held among the reviewers to address any questions, clarify ambiguities, and resolve discrepancies through consensus.

To ensure the accuracy and completeness of the extracted data, a random sample of studies was cross-checked by different reviewers to assess the agreement and identify any potential discrepancies. This process helped enhance the reliability and validity of the extracted data. The extracted data were then compiled and collated into a master database or spreadsheet by implementing a well-defined data extraction protocol with two reviewers. 

Risk of Bias Assessment

The bias assessment was conducted using the Newcastle-Ottawa Scale (NOS) tool for assessing the quality of non-randomised studies [12]. The NOS tool is widely used for evaluating methodological quality in observational studies. It aims to evaluate the quality of non-randomized studies, including cohort and case-control studies, by assigning stars based on three key domains: selection of study groups, comparability of groups, and ascertainment of either the exposure or outcome of interest. The scale provides a structured method for reviewers to appraise the methodological quality of included studies and consider their potential impact on the overall findings of a systematic review. Higher star ratings on the NOS indicate a lower risk of bias and higher methodological quality in the assessed observational studies.

Results

**Figure 1:**
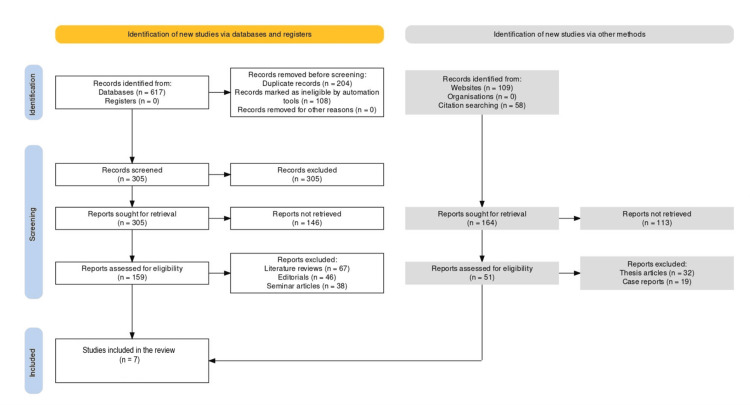
Flow chart of the studies included

The selection of studies included is presented in Figure 1. Table 2 presents key information on seven studies [13-19] on the association between oral microbiota and oral leukoplakia included in the systematic review. Each study is identified by a study ID, along with details such as the year of publication, country where the study was conducted, sample size (n), gender ratio, and mean age in years.

**Table 2:** Demographic characteristics of the studies involved

Study	Year	Country	Sample size (n)	Gender ratio	Mean age (in years)
Amer et al. [13]	2017	Ireland	68	Unspecified	55.45
Gopinath et al. [14]	2020	India	74	Unspecified	Unspecified
Hashimoto et al. [15]	2019	Japan	16	25% females	28-80 (range)
Herreros et al. [16]	2021	Spain	15	60% females	70.5
Kazanowska et al. [17]	2015	Poland	166	64.45% females	Unspecified
Mok et al. [18]	2017	Malaysia	27	63% females	>20
Shridhar et al. [19]	2021	India	91	11% females	45.15

Among the included studies, Amer et al. [13], Gopinath et al. [14], Hashimoto et al. [15], Herreros et al. [16], Kazanowska et al. [17], Mok et al. [18], and Shridhar et al. [19] examined the relationship between oral microbiota and leukoplakia using different methodologies and assessments. The studies varied in terms of the specific microbial species or taxa assessed within the leukoplakia patient population. For example, Amer et al. [13] identified *Campylobacter, Fusobacterium*, and *Leptotrichia* in leukoplakia patients, while Gopinath et al. [14] reported the presence of *Megaspheara, Enterobacteria, Prevotella, Porphyromonas, Rothia, Salmonella, Streptococcus,* and *Fusobacterium*. Hashimoto et al. [15] found changes in the salivary microbiome, with *Bacteroidetes, Solobacterium*, and *Streptococcus* being implicated in leukoplakia. Moreover, the studies differed in their follow-up periods, ranging from unspecified durations to up to 10 years. This variability in follow-up duration might impact the interpretation of the results and the ability to assess the long-term effects of the oral microbiota on leukoplakia development or progression. Comparing the studies, it is evident that there is heterogeneity in the methodologies used, including the gene and its region assessed for microbial analysis. Additionally, there is variation in the reported gender ratios and mean ages of the study participants, which may reflect differences in population demographics and geographical contexts.

Table 3 displays the findings from multiple studies investigating the association between oral microbiota and oral leukoplakia. The studies employed various protocols, including comparative observational, cross-sectional, and case-control designs, and assessed different groups of participants, such as leukoplakia patients, healthy controls, and patients with premalignant conditions or oral cancer. The studies used different genes and their specific regions for microbial assessment, such as 16S rRNA (V1-V2, V3-V4, and V4 regions), 16S rDNA (V6-V9 regions), and *Helicobacter pylori (H. pylori)* DNA. They identified various microbial species or taxa associated with leukoplakia, including *Campylobacter, Fusobacterium, Leptotrichia, Megaspheara, Enterobacteria, Prevotella, Porphyromonas, Rothia, Salmonella, Streptococcus, Bacteroidetes, Solobacterium, Saccharibacteria, Haemophilus, Bacillus, H. pylori, Porphyromonas gingivalis, Fusobacterium nucleatum, Prevotella intermedia,* and several others. In terms of follow-up periods, some studies had unspecified durations, while others spanned up to 10 years or six months.

**Table 3:** Different variables related to leukoplakia and their effect on the oral microbiome as observed in the selected papers

Study	Protocol	Groups assessed	Gene and its region assessed	Microbial species assessed in leukoplakia patients	Follow-up period	Inference assessed
Amer et al. [13]	Comparative observational	36 leukoplakia patients and 32 healthy controls	16S rRNA (V1-V2 region)	Campylobacter, Fusobacterium, Leptotrichia	Unspecified	Patients with leukoplakia displayed an altered oral microbiota that resembled the oral microbiome frequently observed in colorectal cancer.
Gopinath et al. [14]	Cross-sectional	20 leucoplakia patients, 31 oral cancer cases, and 23 healthy controls	16S rRNA (V3-V4 region)	Megaspheara, Enterobacteria, Prevotella, Porphyromonas, Rothia, Salmonella, Streptococcus, Fusobacterium.	Unspecified	The oral microbiomes of patients with leukoplakia and oral cancer showed significant overlap.
Hashimoto et al. [15]	Comparative observational	6 leukoplakia patients, 6 oral squamous cell carcinoma (OSCC) cases, and 4 healthy controls	16S rRNA (V4 region)	Bacteroidetes, Solobacterium, Streptococcus	Unspecified	Patients with leukoplakia exhibited several changes in their salivary microbiome as compared to the healthy controls.
Herreros et al. [16]	Comparative observational	10 leukoplakia patients and 5 healthy individuals	16S rRNA (V3-V4 region)	Campylobacter jejuni, Oribacterium sp., Porphyromonas, Tannerella Eubacterium sp.	10 years	Leukoplakia patients and healthy patients had dramatically different oral microbial diversity and composition.
Kazanowska-Dygdała et al. [17]	Comparative observational	54 leukoplakia patients, 72 with oral lichen planus (OLP) lesions, and 40 healthy controls	*Helicobacter pylori (H. pylori) *DNA	H. pylori	Unspecified	It was observed that leukoplakia might be associated with *H. pylori *presence in the oral microbiome.
Mok et al. [18]	Comparative observational	9 oral cancer patients, 9 patients afflicted with premalignant condition, and 9 healthy controls	16S rDNA (V6-V9 regions)	Streptococcus, Veillonella, Gemella, Granulicatella, Neisseria, Haemophilus, Selenomonas, Fusobacterium, Leptotrichia, Prevotella, Porphyromonas, Lachnoanaerobaculum	Unspecified	The oral microbiome of oral cancer-afflicted patients and patients afflicted with oral premalignant conditions showed somewhat of an overlap.
Shridhar et al. [19]	Case-control	22 leukoplakia patients and 69 healthy controls	Microbial DNA	Porphyromonas gingivalis, Fusobacterium nucleatum, Prevotella intermedia	Six months	Specific pathogen levels were more significantly connected with leukoplakia cases compared to controls.

Overall Inference

**Figure 2:**
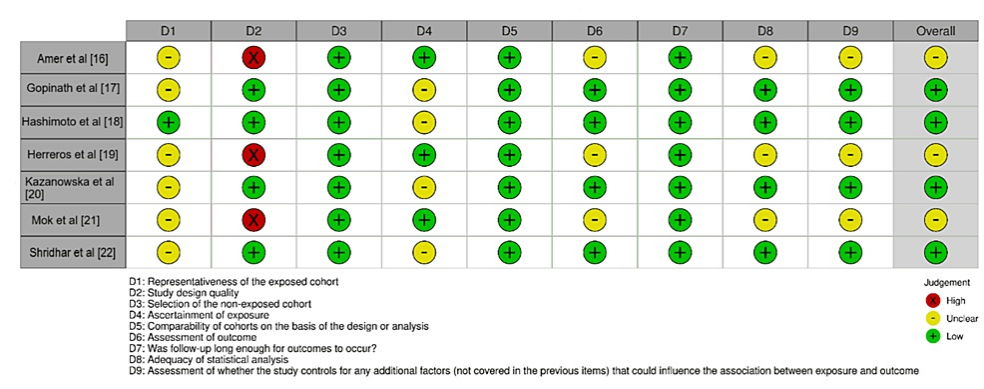
Risk of bias of included studies

Leukoplakia patients and healthy individuals had dramatically different oral microbial diversity and composition. The findings revealed several interesting associations. For example, patients with leukoplakia displayed an altered oral microbiota resembling that frequently observed in colorectal cancer (Amer et al. [13]). Leukoplakia was found to be associated with *H. pylori* presence in the oral microbiome (Kazanowska-Dygdała et al. [17]). Specific pathogen levels were more significantly connected with leukoplakia cases compared to controls. These findings highlight the complex relationship between the oral microbiota and leukoplakia, with variations in the identified microbial species and their associations with the disease. The studies provide insights into potential microbial markers and altercations in the oral microbiome that may be involved in the development and progression of leukoplakia. However, further research is needed to validate these observations, address the limitations of the studies, and elucidate the underlying mechanisms linking the oral microbiota and leukoplakia. The risk of bias of the included studies was moderate as seen in Figure 2. 

Discussion

The findings of this review hold significant implications for both clinical practice and future research. Firstly, the study provides evidence supporting the role of oral microbiota in the development and progression of leukoplakia. By identifying specific microbial species and alterations in the oral microbiome of leukoplakia patients, these findings contribute to a deeper understanding of the disease mechanism and potential biomarkers for early detection and risk assessment. In a clinical context, the identification of an altered oral microbiota resembling that of colorectal cancer in leukoplakia patients suggests potential links between oral and systemic health [13]. This knowledge could inform healthcare professionals about the importance of regular oral health assessments and monitoring of microbial changes in patients with leukoplakia. Furthermore, the overlap observed in the microbiomes of leukoplakic patients and oral cancer cases highlights the potential for shared microbial markers and warrants further investigation into their diagnostic and prognostic significance. The identification of *Campylobacter, Fusobacterium, Leptotrichia, Bacteroidetes, Solobacterium, Streptococcus*, and other microbial species in leukoplakia patients sheds light on the potential microbial etiology and pathogenesis of the condition.

The study by Amer A et al. demonstrated Leukoplakia cohorts had more fungi and fewer *Firmicutes *at p < 0.01. Candidal colonization was also higher in leukoplakia patients (p = 0.025). Clusters of *Fusobacterium, Leptotrichia*, and *Campylobacter* species were highly concentrated (p < 0.01), similar to colorectal tumors [13]. Lingual location demonstrated a higher amount of *Rothia mucilaginosa, Leptotrichia spp.*, and *Campylobacter concisus*. Gopinath et al. found that leukoplakia and oral cancer shared similar bacteria but were different from healthy controls. The distinction noted was by 14 taxa from *Megaspheara*, unclassified enterobacteria, *Prevotella, Porphyromonas, Rothia, Salmonella, Streptococcus*, and *Fusobacterium. Megasphaera*, unclassified *Enterobacteriae, Salmonella, and Prevotella* were the most distinguishable genera between leukoplakia and oral cancer [14]. Hashimoto et al. categorized 448 operational taxonomic units from libraries into 133 genera, 69 families, 41 orders, 26 classes, and 12 phyla. The oral squamous cell carcinoma (OSCC) group has more *Bacteroidetes* and *Solobacterium* than the oral leukoplakia group, but less *Streptococcus *[15]. Kazanowska-Dygdała et al. found leukoplakia in 88% and oral lichen planus (OLP in 93% of buccal mucosa lesions. Around 20% of leukoplakia and 23% of OLP patients have *H. pylori* DNA [17]. The core oral microbiome in Mok et al. showed the dominance of *Firmicutes* and *Streptococcus *[18].

Three of the six most prevalent oral phyla showed significantly different abundances in oral leukoplakia compared to healthy mucosa from the same patients, according to one of the studies that was selected for this review [13]. Similar findings were assessed in another study, which discovered around 40 distinct species in leukoplakia patients as opposed to 20 species in healthy individuals, with eight species being concomitantly present [20]. Given that it could be impacted by periodontal disease or dental caries, our findings call into doubt the reliability of whole-mouth samples in cases of oral leukoplakia [14]. The microbiota composition of leukoplakia and healthy controls was also found to be significantly different in previous saliva-based research [14-15,20-21]. Only a few studies have found any differences in the diversity of the oral microbiome between leukoplakia patients and healthy controls in terms of microbial density [13], reduction [17], and similarity [14,18]. The same two groups have frequently reported differing degrees of diversity [13-14,20]. However, in some cases, the results were challenging to understand due to differences in the target region inside the mouth cavity or the analysis technique.

The systematic review is not without limitations. The first limitation is the lack of standardized protocols for assessing oral microbiota. The studies utilized different gene regions and techniques for microbial analysis, which may affect the comparability and generalizability of the results. Additionally, the variation in the follow-up periods among the studies limits the understanding of the long-term dynamics of oral microbiota in relation to leukoplakia. Furthermore, the limited demographic information provided in some studies, such as the unspecified gender ratios and mean ages, restricts the ability to conduct subgroup analyses or assess potential confounding factors. The lack of detailed information on other relevant factors, such as smoking status, oral hygiene practices, or comorbidities, also limits the comprehensive assessment. Moreover, the majority of the included studies did not provide information on the specific treatment or management strategies for leukoplakia patients. This omission restricts the ability to evaluate the potential role of interventions on the oral microbiota and the subsequent impact on leukoplakia progression or resolution.

## Conclusions

The included studies in this review shed light on specific microbial species and alterations in the oral microbiome of leukoplakia patients, highlighting potential biomarkers and offering a deeper understanding of the disease mechanism. The findings suggest the existence of an altered oral microbiota resembling that of colorectal cancer in leukoplakia patients, indicating possible links between oral and systemic health. Additionally, the significant overlap observed in the oral microbiomes of leukoplakia patients and oral cancer cases underscores the need for further investigation into shared microbial markers and their diagnostic and prognostic implications. Moving forward, addressing the identified research gaps and standardizing methodologies will strengthen our understanding of the complex relationship between oral microbiota and oral leukoplakia, ultimately leading to improved preventive and therapeutic approaches in oral healthcare.
